# Publicly Accessible Large Language Model Responses to Frequently Asked Questions About Spondylodiscitis: Preliminary Expert Evaluation

**DOI:** 10.2196/90364

**Published:** 2026-07-16

**Authors:** Melanie Ardelt, David Schiffelholz, Siegmund Lang, Josina Straub, Sonja Häckel, Nicolas von der Hoeh, Marc Dreimann, Jonathan Neuhoff, Sebastian Siller, Denis Bratelj, Volker Alt, Dietmar Dammerer, Jonas Krueckel

**Affiliations:** 1 Department of Orthopaedics and Traumatology, University Hospital Krems – NOE LGA, Karl Landsteiner University, Mitterweg 10, Krems, 3500, Austria, 43 2732 9004-0; 2 Center for Regenerative Medicine, Department for Health Sciences, Medicine and Research, University for Continuing Education Krems, Krems, Austria; 3 Department of Trauma Surgery, University Hospital Regensburg, Regensburg, Germany; 4 Department of Orthopaedic Surgery and Traumatology, Inselspital, Bern University Hospital, Bern, Switzerland; 5 Department of Orthopaedic, Trauma and Plastic Surgery, University Hospital Leipzig, Leipzig, Germany; 6 Spine Center for Neuroorthopaedics, Spinal Cord Injuries, and Scoliosis, RKH Orthopaedic Clinic Markgröningen, Markgröningen, Germany; 7 Center for Spinal Surgery and Neurotraumatology, Berufsgenossenschaftliche Unfallklinik Frankfurt am Main, Frankfurt, Germany; 8 Department of Neurosurgery, University Hospital of Regensburg, Regensburg, Germany; 9 Spine Surgery, Swiss Paraplegic Center, Nottwil, Switzerland

**Keywords:** spondylodiscitis, artificial intelligence, AI, large language models, spine surgeons, patient education

## Abstract

**Background:**

Patients increasingly use large language models (LLMs) to obtain medical information, but the quality of LLM-generated information on complex spinal infections such as spondylodiscitis remains uncertain. Existing evaluations in spine surgery have mainly addressed degenerative conditions or surgical procedures, and disease-specific data for spondylodiscitis are limited.

**Objective:**

This preliminary study evaluated spine surgeons’ ratings of single-turn LLM responses to 10 author-curated frequently asked questions (FAQs) about spondylodiscitis and compared answer sets generated from GPT-4, GPT-4o, and Google Gemini web interfaces under the authors’ implemented prompting conditions.

**Methods:**

A pool of patient-oriented questions was generated through a chronological workflow including publicly available FAQ sources, PubMed-informed terminology review, Google Trends topic checking, and LLM-generated candidate questions. Duplicate and semantically overlapping questions were removed, the remaining questions were grouped into thematic categories, and 10 final FAQ-style prompts were synthesized. Each prompt was submitted once to the publicly accessible web interfaces of GPT-4, GPT-4o, and Google Gemini. The study was interpreted as an expert evaluation of the resulting answer sets. Seven blinded board-certified spine surgeons rated the responses using a 4-level rating system ranging from excellent to unsatisfactory and additionally assessed comprehensiveness, clarity, empathy, and appropriateness of length. Descriptive statistics and nonparametric comparisons were performed. Interrater reliability was assessed using the intraclass correlation coefficient.

**Results:**

Across all responses, 38.6% (81/210) were rated as excellent, 39% (82/210) as satisfactory with minimal clarification needed, 16.7% (35/210) as satisfactory with moderate clarification needed, and 5.7% (12/210) as unsatisfactory. The most common reason for necessary clarification was insufficient information (58/141, 41.1%), followed by language-related issues (21/141, 14.9%) and overly detailed responses (18/141, 12.8%). The complication-related question received the highest mean rating (3.4/5), whereas treatment- and prognosis-related questions received lower ratings (2.7/5 and 2.9/5). Median overall ratings did not differ significantly among the 3 evaluated LLMs. Spine surgeons reported a generally positive attitude toward artificial intelligence–supported patient information but expressed remaining uncertainty regarding reliability and direct patient-physician communication.

**Conclusions:**

In this preliminary expert evaluation, selected publicly accessible LLM web interfaces generated mostly satisfactory responses to author-curated spondylodiscitis FAQs. The findings reflect outputs produced at the time of access under the authors’ implemented prompting conditions. LLMs may support patient education only with clinician oversight. Future research should explore advanced, domain-specific models to further improve the quality of communication between clinicians and patients.

## Introduction

Spondylodiscitis is an infectious inflammatory condition affecting the intervertebral disc and vertebral bodies, associated with substantial morbidity, prolonged treatment courses, and potential neurological complications [1]. Although relatively rare, accounting for only 3% to 5% of all infectious bone diseases, its incidence has been rising in parallel with an aging population [2-4]. In Germany, absolute case numbers increased from 6886 in 2010 to 9753 in 2020 [2]. Spondylodiscitis carries high morbidity and an in-hospital mortality rate of 17.2% within the first year after diagnosis [5]. The growing number of spondylodiscitis cases remains a major challenge for health care providers globally [6]. Delayed diagnosis and treatment may lead to irreversible neurological deficits, spinal deformity, chronic pain, or sepsis [7,8]. Standard treatment usually involves long-term antibiotic therapy and, in selected cases, surgical intervention [7].

Due to the severity and clinical complexity of spondylodiscitis, many patients and their relatives seek additional information beyond clinical consultations. In the digital era, online health platforms have become important sources of medical information, influencing patients’ understanding, expectations, and health care decisions [8]. While online content may facilitate patient engagement, it is often inconsistent, incomplete, or overly complex, potentially leading to misunderstanding, anxiety, or misinformation [9,10]. As a result, many patients turn to the internet for orthopedic health information to better understand their diagnosis and support informed decision-making [8,11].

Large language models (LLMs), such as ChatGPT and Google Gemini, have recently emerged as tools capable of generating accessible health information [12,13]. LLMs demonstrate potential for patient education by delivering fast and individualized information, and they have shown promising results in different medical domains [14]. Validations of LLM-generated patient information already exist in orthopedic topics such as lumbar disc herniation, lumbar spine fusion surgery, and total knee arthroplasty [15-17]. However, the performance of LLMs specifically in the context of spondylodiscitis—a condition that requires nuanced understanding of infection management, spinal stability, and neurological risk—has not yet been systematically evaluated.

This preliminary study evaluated spine surgeons’ ratings of single-turn LLM responses to 10 author-curated frequently asked questions (FAQs) about spondylodiscitis and compared answer sets generated from GPT-4, GPT-4o, and Google Gemini web interfaces under the authors’ implemented prompting conditions.

## Methods

### Identification of Relevant FAQs

To identify relevant patient-oriented FAQs about spondylodiscitis, a chronological, multisource workflow was applied. Google and PubMed searches were performed on September 14, 2024, in Regensburg, Germany, using English-language search settings and Google Chrome in incognito mode to reduce personalization effects. The Google search term was “FAQ AND spondylodiscitis.” PubMed was used to verify disease-related terminology and clinical topic relevance rather than to extract lay FAQ wording. The first 20 Google results were screened according to the predefined inclusion and exclusion criteria (Textbox 1). Two websites fulfilled the eligibility criteria and contributed patient-oriented FAQ or question and answer content to the initial pool [18,19]. Google Trends was additionally reviewed using the keyword “spondylodiscitis” to identify high-interest topic areas and to support thematic grouping rather than to generate verbatim final questions [20].

To broaden the candidate question pool, GPT-4o and Gemini were each queried with the prompt: “Suggest a list of the 20 most frequently asked patient questions about spondylodiscitis.” Questions identified from eligible FAQ websites and LLM-generated candidate lists were entered into a single spreadsheet. Duplicates and questions with closely overlapping meaning were removed by author consensus. This process resulted in 50 unique candidate questions. Source attribution and the websites contributing questions are provided in Table S1 in Multimedia Appendix 1.

Textbox 1. **Inclusion and exclusion criteria for questions.**

**Inclusion criteria**

- Published after January 1, 2017.
- Published in English language.
- Information presented in frequently asked question or question and answer sections.

**Exclusion criteria**

- Nongeneralizable information, for example, provide or implant specific details.
- Emphasis on nonspine-surgical aspects, for example, anesthesiology information.

**Figure 1.**
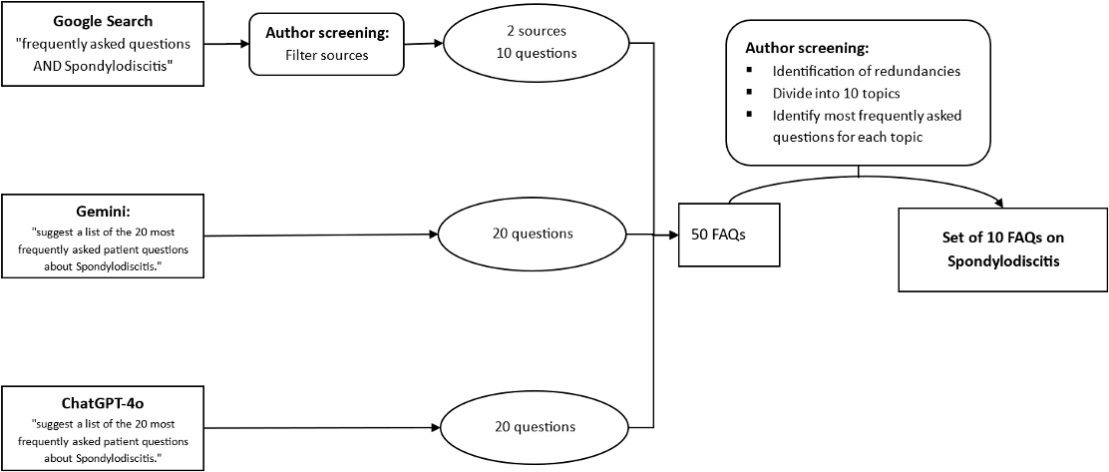
Flowchart of frequently asked questions (FAQs) on spondylodiscitis.

The 50 unique candidate questions were then assigned to 10 predefined thematic categories representing distinct aspects of spondylodiscitis: definition and general understanding, causes and risk factors, symptoms and detection, diagnosis and differential diagnosis, treatment options, management and recovery, complications and long-term effects, prognosis and recurrence, lifestyle and daily life impact, and severity and prevention (Table 1). Within each category, semantically equivalent questions were grouped together, and the frequency of recurring topics across sources was recorded. For each thematic category, the authors synthesized 1 patient-oriented final FAQ prompt that reflected the most frequently recurring or clinically representative wording. This reduction process yielded 10 final prompts, each corresponding to 1 thematic category and collectively covering clinical, procedural, and outcome-related aspects of spondylodiscitis (Textbox 2, Figure 1). Disagreements regarding categorization or final wording were resolved by discussion and consensus among the authors.

The final 10 author-curated FAQ prompts were then used for LLM response generation, as described in Textbox 2.

**Table 1.** Most frequent questions about spondylodiscitis.

Ranking	Topics	Examples	Intentions	Frequency (questions), n
1	Definition and general understanding	“What is spondylodiscitis?”	Understanding the nature of the illness	2
2	Causes and risk factors	“What is the cause of spondylodiscitis?” and “What are the risk factors for developing spondylodiscitis?”	Exploring the origins and contributing factors of the condition	2
3	Symptoms and detection	“What are the symptoms of spondylodiscitis?”	Identifying the signs for recognizing the disease	1
4	Diagnosis and differential diagnosis	“How is spondylodiscitis diagnosed?” and “Can spondylodiscitis be confused with other conditions?”	Clarifying diagnostic processes and distinguishing from similar conditions	2
5	Treatment options	“How is spondylodiscitis treated?” and “Are there non-surgical treatments for spondylodiscitis?”	Outlining the various medical interventions and therapies available	4
6	Management and recovery	“How long does it take to recover from spondylodiscitis?” and “What medications will I need to take?”	Discussing strategies for effective management and the recovery process	3
7	Complications and long-term effects	“What are the potential complications of spondylodiscitis?” and “Can spondylodiscitis cause permanent damage to my spine?”	Considering potential adverse effects and long-term consequences of the illness	7
8	Prognosis and recurrence	“What is the prognosis for spondylodiscitis?” and “Can spondylodiscitis recur after treatment?”	Evaluating the likely course of the disease and the possibility of recurrence	2
9	Lifestyle and daily life impact	“What lifestyle changes can I make to manage my condition (spondylodiscitis)?” and “Will spondylodiscitis impact my quality of life?”	Assessing the impact on daily activities and lifestyle adjustments	7
10	Severity and prevention	“Can spondylodiscitis be prevented?” and “Is spondylodiscitis a serious condition?”	Addressing the seriousness of the condition and measures to prevent it	4

Textbox 2. **Top 10 frequently asked questions about spondylodiscitis.**

1. What is spondylodiscitis?
2. What is the cause of spondylodiscitis?
3. What are the symptoms of spondylodiscitis?
4. How is spondylodiscitis diagnosed?
5. How is spondylodiscitis treated?
6. How long does it take to recover from spondylodiscitis?
7. What are the potential complications of spondylodiscitis?
8. What is the prognosis for spondylodiscitis?
9. What lifestyle changes can I make to manage my condition (spondylodiscitis)?
10. Can spondylodiscitis be prevented?

The 10 final questions were submitted to the publicly accessible GPT-4 web interface [21] on September 14, 2024 (answer set #1). The same questions were submitted to the Google Gemini web interface [22], preceded by the following role-based instruction (answer set #2): “Act as a doctor and expert in the field of management of spondylodiscitis in an orthopedic/neurosurgical setting, who is up to date with the latest scientific research and has years of experience counseling patients with empathy and clarity. Provide a comprehensive and easily understandable answer to the following question about spondylodiscitis. Limit your answer to 150 words and focus on the most important aspects to ensure patient information: (...).” Finally, the same 10 questions were submitted to the publicly accessible GPT-4o web interface [21] (answer set #3). For each question, a new chat window was opened to reduce carry-over effects from previous interactions. The FAQ wording was identical across models, but the surrounding prompting conditions were not fully identical because Gemini received an additional role-based instruction and word limit, whereas GPT-4 and GPT-4o were queried with the question alone. Consequently, the results should be interpreted as a comparison of answer sets generated from selected public web interfaces under the authors’ implemented prompting conditions, not as a controlled, fully reproducible head-to-head benchmark of the underlying model families. All generated answers were transcribed verbatim into the study database for further analysis (Multimedia Appendix 1).

### Raters and Rating of LLM Responses

The answer set generated by each LLM was distributed to the raters via the online Google Forms platform. An expert panel of 7 blinded, board-certified spine surgeons in orthopedics or neurosurgery with extensive experience in spondylodiscitis evaluated the chatbot responses. Unaware that the answers were artificial intelligence (AI)–generated, the assessors rated each response using a validated rating system [23]. The panel included surgeons practicing in Germany, Austria, and Switzerland, thereby situating the assessment within a Central European health care context. A previously published rating system was applied to classify the responses as follows: “excellent response not requiring clarification,” “satisfactory requiring minimal clarification,” “satisfactory requiring moderate clarification,” or “unsatisfactory requiring substantial clarification” [20]. In the case of unsatisfaction (partial or total) with the answers, the raters were asked to identify the reason by selecting one of the following categories: (1) “Off topic, the answer is not pertinent to the question,” (2) “Clear mistakes in the answer,” (3) “Too much information, not all necessary,” (4) “Too little information, not enough for an exhaustive answer,” and (5) “Other reasons.” Additionally, the evaluative framework incorporated 4 supplementary questions (Textbox 3). For each of the 3 answer sets, raters indicated their level of agreement with these statements using a 5-point Likert scale ranging from “strongly disagree” to “strongly agree.”

Finally, the raters were presented with 7 inquiries aimed at eliciting their preference for the best set of 3 responses, followed by additional questions designed to collect their general perspective on the use of AI tools in patient care (Textbox 4). These questions were answered using a 5-point Likert scale.

Textbox 3. **Subsequent inquiries for the Likert scale rating.**

- The overall content of all answers is comprehensive and covers all necessary aspects.
- The answers are easy to understand and are communicated clearly.
- The answers address patient concerns empathetically and professionally.
- The overall length and detail of each answer are appropriate for the target audience.

Textbox 4. **Questions for final evaluation.**

1. “In general, have the above responses met your expectations of the performance of currently available LLMs?”
2. “On the basis of your experience with the scored responses above, would you consider integration of LLM or Al-based patient information into any aspect of your clinical practice in the future?”
3. “In your opinion, how could the use of LLMs improve the patient experience, especially in streamlining the information process before and after surgical procedures?”
4. “Do you think the integration of LLMs in health care could alleviate some of the workload on medical staff, particularly in providing initial information to patients?”
5. “How do you foresee the role of Al or LLMs in optimizing the patient-physician relationship and communication, particularly in ensuring patients are well informed and prepared for their surgical procedures?”
6. “What is your general attitude toward the development of Al or LLMs in health care?”

### Statistical Analysis

Descriptive data are reported as absolute values, percentages, means, and SDs. The distribution of response ratings across predefined categories was analyzed using the Wilcoxon signed-rank test. Differences in ratings between GPT-4, GPT-4o, and Google Gemini were examined with the Kruskal-Wallis test, with post hoc Mann-Whitney *U* tests for pairwise comparisons. Interrater reliability was assessed using the intraclass correlation coefficient (ICC) to quantify the degree of agreement among multiple raters evaluating the same items. All statistical analyses were conducted using GraphPad Prism (version 10.1; GraphPad Software Inc). A *P* value of <.05 was considered statistically significant.

### Reporting Guideline

The manuscript was prepared with reference to the STROBE (Strengthening the Reporting of Observational Studies in Epidemiology) checklist for observational studies.

### Ethical Considerations

This study did not involve patients, patient records, identifiable health information, or clinical interventions. The evaluated material consisted of text responses generated by publicly accessible LLM web interfaces. Physician raters assessed deidentified text outputs through an online survey. The study was determined to be exempt from institutional review board review. Participation of raters was voluntary, and completion of the survey was considered consent to participate. No identifiable rater-level data are reported.

## Results

The distribution of ratings for the combined question set across all 3 models is as follows: 38.6% (81/210) of responses were rated as “excellent” and required no clarification, 39% (82/210) as “satisfactory requiring minimal clarification,” 16.7% (35/210) as “satisfactory requiring moderate clarification,” and 5.7% (11/210) as “unsatisfactory requiring substantial clarification.”

For Gemini, 38.6% (21/70) of responses were rated as excellent and required no clarification, 39% (26/70) as satisfactory requiring minimal clarification, 16.7% (14/70) as satisfactory requiring moderate clarification, and 5.7% (9/70) as unsatisfactory requiring substantial clarification. The prompted version of GPT-4o achieved 38.6% (31/70) of responses rated as excellent and not requiring clarification, 39% (30/70) as satisfactory requiring minimal clarification, 16.7% (8/70) as satisfactory requiring moderate clarification, and only 5.7% (1/70) as unsatisfactory requiring substantial clarification. For GPT-4, 41.4% (29/70) of responses were rated as excellent and required no clarification, 37.1% (26/70) as satisfactory requiring minimal clarification, 18.6% (13/70) as satisfactory requiring moderate clarification, and 2.9% (2/70) as unsatisfactory requiring substantial clarification. The median overall rating across the 10 questions did not differ significantly between Google Gemini, GPT-4o, and GPT-4. These per-model comparisons should be interpreted cautiously, as Gemini was queried with an additional role-based instruction and a 150-word limit, whereas GPT-4 and GPT-4o received the question alone.

**Figure 2.**
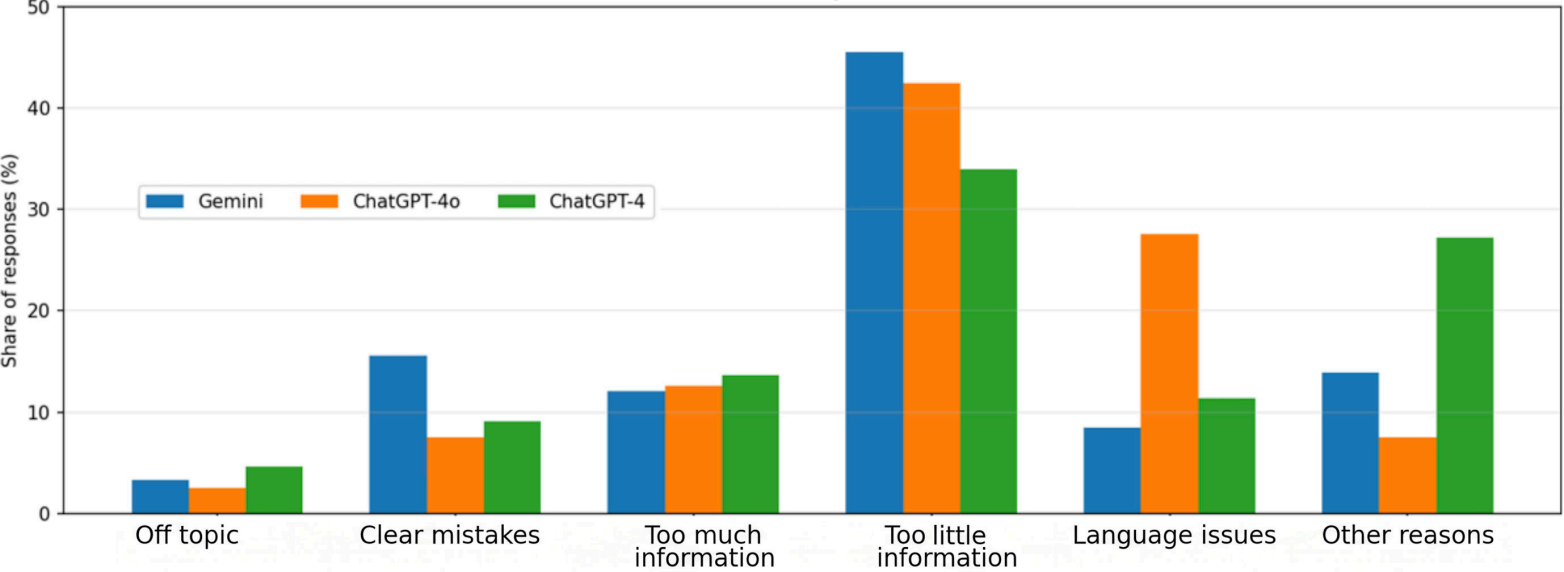
Clarification details for responses generated by large language models.

Among the clarification details, “too little information” was the most frequently cited issue across all models, accounting for 45.6% (26/57) of ratings for Google Gemini, 42.5% (17/40) for GPT-4o, and 34.1% (15/44) for GPT-4. “Language issues,” encompassing unclear phrasing or grammar concerns, were reported in 8.8% (5/57) of evaluations for Google Gemini, 27.5% (11/40) for GPT-4o, and 11.4% (5/44) for GPT-4. “Too much information,” where responses included excessive and unnecessary detail, was noted in 12.3% (7/57) of cases for Google Gemini, 12.5% (5/40) for GPT-4o, and 13.6% (6/44) for GPT-4 (Figure 2). To summarize, the main reasons for dissatisfaction with the responses were as follows: 40.7% provided too little information, 16.3% were attributed to other reasons, 15.9% exhibited language issues, 12.8% provided too much information, 10.8% contained clear mistakes, and 3.5% were rated as off-topic (Figure 2).

**Figure 3.**
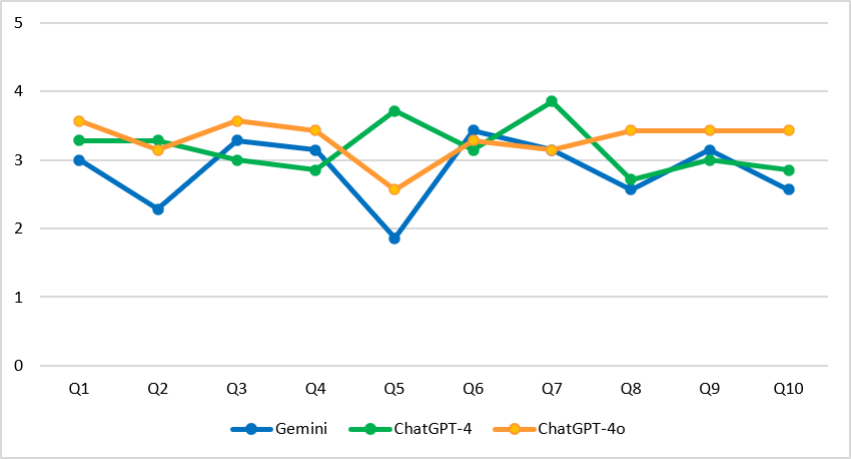
Comparison of mean response ratings for 10 patient questions about spondylodiscitis across 3 large language models. Ratings were assessed on a 5-point Likert scale (1=lowest possible rating and 5=highest possible rating).

On the basis of the mean ratings across all 3 models, question 7 (“What are the potential complications of spondylodiscitis?” 3.4/5 points) received the highest overall score, indicating the best response quality. Question 1 (“What is spondylodiscitis?” 3.3/5), question 3 (“What are the symptoms of spondylodiscitis?” 3.3/5), and question 6 (“How long does it take to recover from spondylodiscitis?” 3.3/5) ranked next, showing similar performance. In contrast, question 5 (“How is spondylodiscitis treated?” 2.7/5) and question 8 (“What is the prognosis for spondylodiscitis?” 2.9/5) received the lowest ratings. The median overall rating across the 10 questions did not differ significantly among Google Gemini, GPT-4o, and GPT-4. Median scores were generally consistent across all models, with only minor variations observed for individual questions (Figure 3).

The overall assessment of AI in health care by the participating spine surgeons was positive. The highest agreement was observed regarding the potential of AI to improve patient experience (4.3/5), integrate into practice (4.3/5), and enhance general attitudes toward AI in health care (4.3/5). Surgeons also believed that AI could contribute to reducing clinical workload (4.0/5). In contrast, expectations of performance (3.7/5) and the ability of AI to optimize patient-physician communication (3.8/5) received slightly lower ratings, indicating remaining uncertainty about AI’s reliability and its role in direct patient interaction.

Across all 30 model-question ratings evaluated by 7 raters, the single-measure ICC was 0.19, indicating low agreement at the individual rater level, whereas the average-measure ICC was 0.62, indicating moderate reliability of the aggregated expert panel rating. Model-specific average-measure ICCs were 0.64 for Gemini, 0.54 for GPT-4, and 0.49 for GPT-4o. These findings suggest that individual ratings varied between experts, but consensus panel averages showed moderate reliability.

## Discussion

### Principal Findings

This preliminary study found that spine surgeons rated single-turn responses from 3 publicly accessible LLM interfaces to author-curated spondylodiscitis FAQs as mostly satisfactory or excellent. The highest ratings were observed for general disease information and potential complications, whereas treatment and prognosis questions received lower ratings. These findings suggest that current LLMs can summarize relatively standardized medical information, but they remain less reliable for questions requiring individualized clinical judgment.

These findings are consistent with previous studies in other spinal conditions, including lumbar disc herniation, spinal cord injury, spine fusion surgery, and adolescent idiopathic scoliosis, where LLM-generated patient information was mostly rated as clinically useful and understandable [15,16,24,25]. For example, in patient education regarding adolescent idiopathic scoliosis, 26% of responses across multiple LLMs were judged as “excellent” [25]. Similarly, studies on lumbar disc herniation reported that 27.2% of ChatGPT responses were classified as excellent and 43.9% as satisfactory with minimal clarification, though a small proportion remained unsatisfactory [26]. In another evaluation focused on acute lumbar disc herniation, ChatGPT delivered mostly accurate and comprehensible patient-focused information but occasionally included incorrect treatment suggestions and did not fully match the content of formal informed consent documents [16]. Supporting these positive trends, a recent scoping review showed that GPT-4 achieved up to 92% accuracy in clinical recommendations, outperforming earlier model versions and exhibiting fewer hallucinations than prior generations [27]. A study evaluating the readability of OrthoInfo spine articles and the impact of LLM-based simplification demonstrated that these models can significantly improve orthopedic patient education by enhancing readability while maintaining acceptable accuracy [28]. Together, these studies underscore that LLMs already offer a promising tool for enhancing patient education in spine care, provided that their output is used with appropriate clinical oversight.

However, performance is not yet consistent across medical contexts. Studies assessing LLM adherence to surgical evidence-based guidelines from the North American Spine Society reported substantial limitations, demonstrating only partial concordance with evidence-based recommendations, particularly when no clear clinical consensus exists [29,30]. A study evaluating their adherence to surgical guidelines found that none of the models—Bard, BingAI, GPT-3.5, or GPT-4—achieved full concordance with the North American Spine Society recommendations. GPT-4 and BingAI reached the highest alignment, each at 60% [30]. Similarly, another study on GPT-4 in spinal metastasis treatment planning found reasonable alignment with surgeons (73%), a clear tendency toward generalized and less actionable recommendations in controversial decisions such as surgery and palliative strategies [31]. Furthermore, another study reported that in situations where no definitive best practices exist, the reliability of LLM-generated recommendations declines substantially [32]. In such contexts, responses were frequently overly general or inconsistent with the literature and, in some instances, even included fabricated data or citations, emphasizing that cautious interpretation and clinical oversight remain essential when using LLMs in ambiguous decision-making scenarios [32].

Promisingly, radiology-focused questions have shown particularly strong results [33-36]. LLM-supported tools have improved accuracy and efficiency in scoring systems such as Spine Instability Neoplastic Score, and models combining imaging features with clinical text already approach performance levels relevant for diagnostic support [37]. Another study found that LLMs passed written board exam sections but failed when image-based reasoning was required [38]. Nevertheless, image-dependent reasoning remains challenging and should be applied with appropriate caution.

As expected, definitions of spondylodiscitis and its possible complications were answered well by the evaluated LLMs, as these aspects are clearly and consistently described in the literature [39-42]. In contrast, questions regarding treatment and prognosis, which depend on numerous individual factors, remain more challenging for LLMs to address. While some studies recommend early surgical intervention, conservative treatment options may also be appropriate depending on the case, resulting in a lack of universally accepted best practices [43,44]. Consistent with this, a previous study demonstrated that GPT-4o showed variable depth in its clinical responses (mean 3.7, SD 0.6), and “red flag” symptoms were missed in 7.1% of cases [45].

Beyond mere information retrieval, the lower scores in the “treatment” and “prognosis” categories in this study suggest that current LLMs struggle with the clinical nuances of spondylodiscitis. Future applications should evolve toward retrieval-augmented generation systems, which integrate verified clinical guidelines to minimize hallucinations and ensure that medical advice remains evidence based [46].

Furthermore, LLMs could enhance shared decision-making by acting as a “consultation coach.” As previously demonstrated, AI can provide high-quality, empathetic responses that help patients structure their concerns [47]. This shift from providing raw data to facilitating a high-quality patient-physician dialogue could significantly reduce the clinical workload while allowing more time for complex, human-centered medical decisions [48].

Overall, current LLMs show promising potential for supporting patient education in spondylodiscitis and other spine conditions, especially when presenting well-established medical knowledge. However, their accuracy still varies, particularly when clinical judgment or individualized treatment decisions are required. Therefore, LLMs should be used carefully in clinical settings. Future improvements should aim to better align with clinical guidelines, reduce incorrect information, stay updated with current evidence, and strengthen their ability to work with different types of medical data to ensure safe use in practice.

### Limitations

This study has several limitations. First, the question set consisted of author-curated FAQ-style prompts derived from publicly available sources and LLM-generated candidate questions. These prompts may not reflect real patient-generated questions, which are often more emotional, fragmented, context dependent, and influenced by varying levels of health literacy. Second, the evaluation was performed exclusively by spine surgeons. Although expert assessment is important for medical accuracy, clinicians cannot fully assess patient comprehension, readability, anxiety, trust, perceived usefulness, or behavioral consequences. Future studies should include patients, relatives, and lay users. Third, each model generated only 1 response per question. LLM outputs may vary across repeated prompts, dates, interface updates, and model versions; therefore, future work should repeat each prompt multiple times and assess output stability. Fourth, the models were accessed through public web portals rather than through controlled application programming interface settings. Consequently, key implementation details and generation parameters were not controllable or fully observable, including exact model version, backend routing, hidden system-level instructions, safety filters, browsing or retrieval status, temperature, maximum output length, and other decoding parameters. This directly limits reproducibility and may affect fairness of comparisons across models. The findings therefore reflect the behavior of the tested public web interfaces on September 14, 2024, rather than stable or fully reproducible performance of the underlying model families. Fifth, the FAQ wording was identical across the models, but the surrounding prompting conditions were not fully identical because Gemini received an additional role-based instruction and a 150-word limit, whereas GPT-4 and GPT-4o were queried with the question alone. This may have influenced response length, clarity, empathy, comprehensiveness, and expert ratings. Accordingly, the study should not be interpreted as a strict head-to-head comparison of model capabilities. Sixth, due to the rapid advancement of LLMs, results may quickly become outdated, as evidenced by frequent model updates and new releases (eg, successive ChatGPT versions). Although 1 study evaluated GPT-4o and GPT-5 using FAQs based on information from the Cleveland Clinic website on selected spine surgeries—specifically transforaminal lumbar interbody fusion, posterior lumbar interbody fusion, lateral lumbar interbody fusion, posterior cervical decompression and fusion, and anterior cervical decompression and fusion—the models demonstrated differing performance characteristics: GPT-4o provided more readable responses overall, whereas GPT-5 generated more reliable citations [49]. Seventh, the number of raters was limited, and the study was situated within a Central European spine surgery context, which may restrict generalizability. Finally, the study did not assess clinical outcomes, real-world use, interactive follow-up questions, safety in individualized scenarios, or workflow effects.

Overall, the participating spine surgeons demonstrated a generally positive attitude toward LLMs, which could have influenced their ratings and resulted in more favorable evaluations. Future studies would benefit from larger and more diverse rater groups, as well as a mixed methods design incorporating physician-validated reference answers and qualitative feedback from both patients and clinicians. Finally, while general-purpose LLMs are widely available, specialized medical models may provide more accurate and focused information, yet remain less accessible to the public, such as ClinicalGPT and Google’s Med-PaLM 2.

### Conclusions

In this preliminary expert-rated evaluation, selected publicly accessible LLM web interfaces generated mostly satisfactory responses to author-curated FAQ-style questions about spondylodiscitis. However, performance was topic dependent: responses to general and complication-related questions were rated more favorably than responses addressing treatment and prognosis. As public web interface settings and prompting conditions were not fully controllable, the results should not be interpreted as a definitive, stable, or fully reproducible head-to-head comparison of GPT-4, GPT-4o, and Google Gemini. These findings support a cautious approach to further investigation of LLMs as adjunctive tools for patient information, but they do not establish real-world patient-facing safety, comprehension, clinical effectiveness, or workflow benefits. LLM-generated information should be used only with clinician oversight, and future studies should incorporate real patient questions, repeated prompting, patient and lay user ratings, readability and safety metrics, controlled generation settings where feasible, and transparent prompt-response datasets.

## Supplementary material

10.2196/90364 Multimedia Appendix 1 Answers and questions for the different large language models.

10.2196/90364 Checklist 1 STROBE checklist.

10.2196/90364 Figure S1.

## Abbreviations

AI: artificial intelligence
FAQ: frequently asked question
LLM: large language model
STROBE: Strengthening the Reporting of Observational Studies in Epidemiology
