# Supplementary material for: Publicly Accessible Large Language Model Responses to Frequently Asked Questions About Spondylodiscitis: Preliminary Expert Evaluation
Source: J Med Internet Res. 2026 Jul 16;28:e90364. doi: 10.2196/90364 (PMC13374826; doi:10.2196/90364)
Supplement: Multimedia Appendix 1 [file jmir-v28-e90364-s001.docx]

Table 1: What is Spondylodiscitis?

| Question:  „What is Spondylodiscitis?" | excellent answer not requiring clarification | satisfactory, requiring minimal clarification | satisfactory, requiring moderate clarification | unsatisfactory, requiring substantial clarification |
| --- | --- | --- | --- | --- |
| ChatGPT-4:  Spondylodiscitis is an infection of the spine that affects both the intervertebral discs (the soft, cushion-like structures between the vertebrae) and the adjacent vertebral bodies, the bones that make up the spine. This condition can be caused by bacteria or fungi, which may reach the spine through the bloodstream from other parts of the body or directly from procedures or surgeries. Symptoms often include severe back pain, fever, and sometimes neurological deficits if the infection compresses spinal nerves.  Diagnosis is typically confirmed through magnetic resonance imaging (MRI), which provides detailed images of the affected structures, and blood tests that indicate infection. Treatment generally involves a combination of long-term antibiotics or antifungal medications and may require surgical intervention to stabilize the spine and relieve pressure on affected nerves. Prompt, effective management is crucial to prevent complications and promote recovery. |  |  |  |  |
| ChatGPT-4o:  Spondylodiscitis is an infection of the spine that involves both the intervertebral disc and the adjacent vertebrae. It can be caused by bacteria (most commonly Staphylococcus aureus), fungi, or other microorganisms spreading through the bloodstream or directly from nearby tissues. The condition usually presents with back pain, fever, and sometimes neurological symptoms like weakness or numbness due to spinal cord compression.  Diagnosis typically involves imaging (such as MRI) and blood tests, while treatment often includes a combination of antibiotics or antifungals and, in some cases, surgery to remove infected tissue, stabilize the spine, or decompress nerves.  Early diagnosis and treatment are crucial to prevent complications like spinal deformity or permanent nerve damage. The recovery process can be lengthy, requiring physical rehabilitation and regular follow-up. |  |  |  |  |
| Gemini (Google Bard):  Spondylodiscitis is a serious infection that affects the vertebrae and the intervertebral disc. It's essentially a bacterial or fungal infection that can cause inflammation, pain, and potential nerve compression.  The infection often starts in the bloodstream, spreading to the spine. Symptoms can include severe back pain, fever, chills, and loss of appetite. In severe cases, the infection can lead to spinal abscesses or even spinal collapse.  If you suspect you may have spondylodiscitis, it's crucial to seek medical attention immediately. Early diagnosis and treatment can prevent complications and improve your chances of a full recovery. |  |  |  |  |

Table 2: What is the cause of Spondylodiscitis?

| Question:  "What is the cause of Spondylodiscitis?" | excellent answer not requiring clarification | satisfactory, requiring minimal clarification | satisfactory, requiring moderate clarification | unsatisfactory, requiring substantial clarification |
| --- | --- | --- | --- | --- |
| ChatGPT-4:  Spondylodiscitis is an infection of the spine that primarily affects the intervertebral discs and the adjacent vertebral bodies. It can be caused by a bacterial or fungal organism. The most common bacterial cause is Staphylococcus aureus. The infection typically reaches the spinal structures via the bloodstream from another infected site in the body, although it can also occur after spinal surgery or due to direct infection from a penetrating injury. People with weakened immune systems, such as those with diabetes, those who use intravenous drugs, or those who have recently undergone invasive procedures, are at higher risk. Early diagnosis and treatment are crucial to prevent complications such as abscess formation and spinal instability. |  |  |  |  |
| ChatGPT-4o:  Spondylodiscitis is an infection of the intervertebral disc and adjacent vertebrae, typically caused by bacteria. The most common pathogen is Staphylococcus aureus, but other bacteria such as Escherichia coli and Mycobacterium tuberculosis can also be responsible. The infection usually spreads to the spine through the bloodstream from another site in the body, like the skin, urinary tract, or lungs. Risk factors include a weakened immune system, previous spinal surgery, intravenous drug use, diabetes, and older age. In some cases, it can occur after direct trauma or spinal procedures. Early diagnosis and treatment are critical to prevent complications such as chronic pain or neurological deficits. Treatment typically involves antibiotics, sometimes paired with surgery if there is severe spinal instability, abscess formation, or neurological compromise. |  |  |  |  |
| Gemini (Google Bard):  Spondylodiscitis is a bacterial infection of the intervertebral disc and adjacent vertebrae. It's a serious condition that can lead to significant pain, neurological deficits, and even spinal instability if left untreated.  The most common causes of Spondylodiscitis are:   1. Staphylococcus aureus: This bacteria is a common cause of skin infections, pneumonia, and food poisoning. It can also spread to the bones and joints. 2. Mycobacterium tuberculosis: This bacteria is the cause of tuberculosis, a chronic infectious disease that primarily affects the lungs. In some cases, it can also spread to the bones. 3. Salmonella: This bacteria is often associated with food poisoning. It can also cause infections in other parts of the body, including the bones.   Less common causes include other bacteria, fungi, and parasites. |  |  |  |  |

Table 3: What are the symptoms of Spondylodiscitis?

| Question:  "What are the symptoms of Spondylodiscitis?" | excellent answer not requiring clarification | satisfactory, requiring minimal clarification | satisfactory, requiring moderate clarification | unsatisfactory, requiring substantial clarification |
| --- | --- | --- | --- | --- |
| ChatGPT-4:  Spondylodiscitis, an infection of the vertebral discs and adjacent vertebrae, often presents with back pain that’s typically severe and localized. This pain may worsen with movement or at night, and is not relieved by rest. Patients may also experience fever, though it’s not always present. As the infection progresses, you might notice radiating pain, weakness, or numbness if the infection impacts nearby nerve structures. In some cases, there’s noticeable tenderness over the affected spinal area. Advanced cases can lead to more severe neurological deficits such as changes in reflexes, loss of bowel or bladder control, or even paralysis, depending on the nerves involved. It’s crucial to seek medical attention promptly if these symptoms are experienced, as early diagnosis and treatment can significantly improve outcomes. |  |  |  |  |
| ChatGPT-4o:  Spondylodiscitis, an infection of the intervertebral disc and adjacent vertebrae, presents with a variety of symptoms, typically developing over time. The most common symptom is severe, persistent back pain that worsens with movement but is not relieved by rest. This pain is often localized and can radiate to the limbs if nerve roots are involved.  Other key symptoms include fever, chills, and night sweats, though these are not always present, especially in older adults. Patients may also experience difficulty walking, muscle weakness, or neurological deficits if the infection compresses the spinal cord or nerves. In severe cases, weight loss, fatigue, and general malaise can occur.  If you notice these symptoms, especially if combined with recent infections, immunosuppression, or recent spinal procedures, seek medical attention promptly. Early diagnosis and treatment are crucial for preventing complications like paralysis or chronic pain. |  |  |  |  |
| Gemini (Google Bard):  Spondylodiscitis, an infection of the intervertebral disc and adjacent vertebrae, often presents with a combination of symptoms:   1. Back pain: This is typically severe, constant, and may worsen with activity. 2. Fever: Although not always present, especially in older adults, fever is a common symptom. 3. Neurological symptoms: These can include numbness, tingling, or weakness in the legs, as well as changes in bowel or bladder function. 4. Night sweats: Excessive sweating during sleep can be another symptom. 5. Weight loss: Unintentional weight loss may occur due to the illness.   If you experience these symptoms, it's important to seek medical attention promptly. Early diagnosis and treatment can help prevent complications and improve your recovery. |  |  |  |  |

Table 4: How is Spondylodiscitis diagnosed?

| Question:  "How is Spondylodiscitis diagnosed?" | excellent answer not requiring clarification | satisfactory, requiring minimal clarification | satisfactory, requiring moderate clarification | unsatisfactory, requiring substantial clarification |
| --- | --- | --- | --- | --- |
| ChatGPT-4:  Spondylodiscitis is diagnosed primarily through a combination of clinical evaluation and imaging studies. Initially, your doctor will discuss your symptoms and medical history, focusing on any recent infections or procedures. Key symptoms typically include back pain, fever, and possibly neurological deficits.  Imaging tests play a crucial role. An MRI (Magnetic Resonance Imaging) is the most effective tool, providing detailed images of the bones and surrounding soft tissues, and can identify inflammation or abscesses. If MRI is not available or feasible, a CT scan may be used, though it’s less sensitive.  Blood tests are also important; they can detect inflammation and infection markers such as elevated C-reactive protein (CRP) and erythrocyte sedimentation rate (ESR). In some cases, a biopsy or microbiological tests may be needed to identify the specific infectious agent, guiding targeted treatment. |  |  |  |  |
| ChatGPT-4o:  Spondylodiscitis is diagnosed through a combination of clinical evaluation, imaging, and laboratory tests.  Doctors begin by reviewing the patient’s symptoms, which often include back pain, fever, and neurological issues like numbness or weakness. They will also ask about recent infections, surgeries, or underlying conditions like diabetes.  Key imaging tests include MRI, which is the gold standard, as it clearly shows inflammation in the vertebrae and discs. CT scans or X-rays can also help, but are less sensitive.  Blood tests, including inflammatory markers (CRP, ESR), and blood cultures, help identify infection. In some cases, a biopsy or aspiration of the affected area is done to determine the specific organism causing the infection, guiding antibiotic treatment.  Early diagnosis is crucial to prevent complications like abscesses or permanent nerve damage. A multidisciplinary approach involving orthopedics, neurosurgery, and infectious disease specialists ensures optimal care. |  |  |  |  |
| Gemini (Google Bard):  Spondylodiscitis, an infection of the vertebrae and the disc between them, is typically diagnosed through a combination of clinical symptoms and diagnostic tests.   1. Clinical Symptoms: These often include back pain, fever, night sweats, weight loss, and neurological symptoms like weakness or numbness. 2. Laboratory Tests: Blood tests can reveal elevated white blood cell count and markers of inflammation. 3. Imaging Studies: X-rays may show signs of bone destruction, but MRI is often the most sensitive test for detecting inflammation and abscess formation in the spine. CT scans can be used to guide biopsies or drainage procedures. 4. Bone Biopsy: In some cases, a bone biopsy may be necessary to confirm the diagnosis and identify the specific type of bacteria causing the infection. |  |  |  |  |

Table 5: How is Spondylodiscitis treated?

| Question:  "How is Spondylodiscitis treated?" | excellent answer not requiring clarification | satisfactory, requiring minimal clarification | satisfactory, requiring moderate clarification | unsatisfactory, requiring substantial clarification |
| --- | --- | --- | --- | --- |
| ChatGPT-4:  Spondylodiscitis, an infection of the vertebral discs and adjacent vertebrae, requires prompt and thorough treatment to prevent complications. The cornerstone of treatment is prolonged antibiotic therapy, often tailored to the specific bacteria causing the infection, which is typically identified through blood tests or a biopsy. The duration of antibiotic treatment can range from six weeks to several months, depending on the severity and response to therapy.  In some cases, especially when there is spinal instability or significant nerve compression, surgical intervention may be necessary. Surgical options include debridement (removal of infected tissue), decompression of neural elements, and possibly spinal fusion to stabilize the affected segment.  Supportive care, including pain management and possibly physical therapy, plays a vital role in recovery, aiming to maintain muscle strength and mobility while the spine heals. Regular follow-up with imaging and clinical evaluation is crucial to monitor the treatment response and adjust the plan as needed. |  |  |  |  |
| ChatGPT-4o:  Spondylodiscitis, an infection of the vertebrae and intervertebral discs, requires prompt and comprehensive treatment to avoid complications. The primary approach includes antibiotic therapy tailored to the specific bacteria causing the infection, usually administered intravenously for 4–6 weeks, followed by oral antibiotics.  In some cases, surgical intervention may be needed, especially if there is spinal instability, abscess formation, or neurological impairment. Surgery aims to remove infected tissue, decompress the spinal cord, and stabilize the spine if necessary.  Pain management and immobilization with a brace can help alleviate discomfort and support healing. Rehabilitation through physiotherapy is crucial once the infection is controlled to restore mobility and strength.  Throughout treatment, close monitoring with blood tests, imaging, and clinical assessments is essential to ensure recovery and prevent recurrence. Early diagnosis and a multidisciplinary approach between orthopedics, neurosurgery, and infectious disease specialists improve outcomes. |  |  |  |  |
| Gemini (Google Bard):  Spondylodiscitis, an infection of the spine, typically requires a combination of treatments to effectively address both the infection and the structural damage it causes.  Key treatments often include:   1. Antibiotics: These are essential to combat the infection. The specific type and duration of antibiotic treatment depend on the identified bacteria causing the infection. 2. Bed rest: Initially, bed rest is often prescribed to immobilize the spine and reduce pain. 3. Pain management: Medications can help alleviate pain associated with the condition. 4. Bracing or immobilization: In some cases, a brace or other immobilizing device might be used to support the spine. 5. Surgery: If the infection is severe or if there's significant structural damage, surgery may be necessary. This could involve draining an abscess, removing infected tissue, or stabilizing the spine with implants.   The specific treatment plan will be tailored to the individual patient's condition and may evolve as the infection progresses. Regular follow-up with healthcare professionals is crucial to monitor treatment effectiveness and address any complications. |  |  |  |  |

Table 6: How long does it take to recover from Spondylodiscitis?

| Question:  "How long does it take to recover from Spondylodiscitis?" | excellent answer not requiring clarification | satisfactory, requiring minimal clarification | satisfactory, requiring moderate clarification | unsatisfactory, requiring substantial clarification |
| --- | --- | --- | --- | --- |
| ChatGPT-4:  Recovery from spondylodiscitis, an infection of the spinal discs and adjacent vertebrae, generally varies depending on the severity of the infection and the patient’s overall health. Typically, treatment involves long-term antibiotic therapy, which may last from 6 to 12 weeks, coupled with immobilization of the spine and sometimes surgical intervention if necessary. After initiating treatment, clinical improvement can often be seen within a few weeks, but complete recovery can take several months. Regular follow-ups with your healthcare provider, including blood tests and imaging, are crucial to monitor progress and adapt treatment as needed. Achieving full recovery is a gradual process, and it's important to maintain close communication with your medical team throughout your treatment journey to ensure the best outcomes. |  |  |  |  |
| ChatGPT-4o:  Recovery from spondylodiscitis, an infection of the spine, varies based on severity, patient health, and treatment. Typically, recovery takes 3 to 6 months with proper treatment, which often includes long-term antibiotics (usually 6-12 weeks), sometimes delivered intravenously initially, followed by oral antibiotics. If the infection has caused significant damage, surgery may be required to stabilize the spine or remove infected tissue.  Pain management and physical rehabilitation are key components of recovery, as patients often experience back pain and reduced mobility. Improvement in symptoms like pain and inflammation is usually seen within the first weeks of treatment, but full recovery, including regaining mobility, can take months.  Close monitoring with regular medical follow-ups is essential to ensure the infection resolves and complications are avoided. Early treatment improves recovery outcomes and reduces the risk of lasting spinal damage. |  |  |  |  |
| Gemini (Google Bard):  Recovery from Spondylodiscitis is a gradual process that can vary significantly from person to person. The duration of recovery depends on several factors, including the severity of the infection, the type of treatment received, and the individual's overall health.  Generally, recovery from Spondylodiscitis can take several weeks to months. Initial treatment, which often involves antibiotics and possibly surgery, aims to control the infection and prevent further damage to the spine. Once the infection is under control, rehabilitation therapy can help restore strength and mobility.  It's important to note that full recovery may not be immediate. Some individuals may experience lingering symptoms or limitations, even after the infection is resolved. Regular follow-up with your healthcare provider is essential to monitor your progress and address any ongoing concerns. |  |  |  |  |

Table 7: What are the potential complications of Spondylodiscitis?

| Question:  "What are the potential complications of Spondylodiscitis?" | excellent answer not requiring clarification | satisfactory, requiring minimal clarification | satisfactory, requiring moderate clarification | unsatisfactory, requiring substantial clarification |
| --- | --- | --- | --- | --- |
| ChatGPT-4:  Spondylodiscitis, an infection of the spinal discs and adjacent vertebrae, can lead to several serious complications if not properly managed. The most significant complications include:  1. Abscess Formation: Pus can accumulate in or around the spinal column, potentially compressing spinal nerves.  2. Spinal Instability: The infection can weaken the vertebrae, leading to structural changes or collapse, causing severe pain and deformity.  3. Neurological Impairment: Compression or damage to spinal nerves or the spinal cord can result in weakness, numbness, or even paralysis in the limbs.  4. Systemic Infection: The infection can spread to other parts of the body, becoming more difficult to treat, especially in cases of delayed diagnosis or treatment.  Prompt and effective treatment is essential to prevent these complications, typically involving antibiotics and sometimes surgical intervention. |  |  |  |  |
| ChatGPT-4o:  Spondylodiscitis, an infection of the intervertebral disc and adjacent vertebrae, can lead to serious complications if not treated promptly. Key complications include:  1. Abscess Formation: Epidural or paraspinal abscesses may compress the spinal cord or nerve roots, causing pain, weakness, or paralysis.  2. Neurological Deficits: Compression from infection or abscesses can result in permanent nerve damage, leading to sensory loss, muscle weakness, or in severe cases, paralysis.  3. Spinal Instability: Infection may weaken the vertebrae, increasing the risk of fractures or spinal deformity.  4. Sepsi: If the infection spreads to the bloodstream, it can cause life-threatening sepsis.  5. Chronic Pain: Even after treatment, patients may experience long-term back pain or stiffness.  Early diagnosis and treatment (antibiotics, surgery if needed) are critical to prevent these complications. |  |  |  |  |
| Gemini (Google Bard):  Spondylodiscitis, an infection of the intervertebral disc, can lead to several serious complications if left untreated or poorly managed. The most significant risks include:   1. Neurological deficits: In severe cases, the infection can spread to the spinal cord or nerves, leading to weakness, numbness, or even paralysis. 2. Vertebral collapse: The weakened disc and surrounding bone may collapse, resulting in spinal deformity and pain. 3. Abscess formation: Pus-filled abscesses can develop around the infected disc, causing pressure on nearby structures and potentially leading to neurological complications. 4. Sepsis: In rare cases, the infection can spread throughout the body, leading to a life-threatening condition called sepsis.   It's important to seek immediate medical attention if you suspect spondylodiscitis. Early diagnosis and treatment can help prevent these complications and improve your chances of a full recovery. |  |  |  |  |

Table 8: What is the prognosis for Spondylodiscitis?

| Question:  "What is the prognosis for Spondylodiscitis?" | excellent answer not requiring clarification | satisfactory, requiring minimal clarification | satisfactory, requiring moderate clarification | unsatisfactory, requiring substantial clarification |
| --- | --- | --- | --- | --- |
| ChatGPT-4:  Spondylodiscitis, an infection of the spinal discs and adjacent vertebrae, generally has a favorable prognosis when diagnosed early and treated effectively. Treatment usually includes antibiotics for at least 6 to 12 weeks and may also involve immobilization of the spine or surgery if there is significant damage to the spinal structure or neurological complications. The recovery period can vary, depending on the severity of the infection and the patient's overall health. Factors such as age, pre-existing health conditions, and the causative organism can influence outcomes. Adherence to treatment and regular follow-up with healthcare providers are crucial to managing symptoms and preventing complications. With appropriate treatment, most patients can expect significant improvement or complete recovery. |  |  |  |  |
| ChatGPT-4o:  The prognosis for spondylodiscitis depends on early diagnosis and treatment. With prompt intervention, which typically includes antibiotics and sometimes surgery, most patients recover well. However, recovery can take months, and some may experience persistent pain or limited mobility, especially if nerve damage or spinal deformity occurs.  Factors influencing prognosis include the patient's overall health, the presence of complications (like abscesses), and the specific bacteria causing the infection. Older age, delayed treatment, or other chronic conditions (e.g., diabetes) can worsen outcomes.  With timely and appropriate care, infection control is usually effective, and long-term complications are minimized. Regular follow-ups are essential to monitor progress and prevent recurrence. Pain management, physical therapy, and sometimes bracing may be necessary during recovery. |  |  |  |  |
| Gemini (Google Bard):  The prognosis for Spondylodiscitis, a serious infection of the spine, depends on various factors, including the severity of the infection, the patient's overall health, and the effectiveness of treatment.  Generally, with prompt and appropriate treatment, most patients with Spondylodiscitis can make a full recovery. However, the recovery process may take several months. In severe cases, complications such as neurological deficits or spinal instability may occur, requiring more intensive treatment or surgical intervention.  It's crucial to follow your doctor's treatment plan closely and attend all follow-up appointments. Early diagnosis and treatment are key to improving the prognosis and minimizing complications. |  |  |  |  |

Table 9: What lifestyle changes can I make to manage my condition (Spondylodiscitis)?

| Question:  "What lifestyle changes can I make to manage my condition (Spondylodiscitis)?" | excellent answer not requiring clarification | satisfactory, requiring minimal clarification | satisfactory, requiring moderate clarification | unsatisfactory, requiring substantial clarification |
| --- | --- | --- | --- | --- |
| ChatGPT-4:  To manage spondylodiscitis effectively, it's important to focus on lifestyle adjustments that promote spine health and overall well-being. Here are key changes you can make:  1. Maintain a Healthy Weight: Reducing body weight can decrease stress on your spine.  2. Nutrition: Adopt a balanced diet rich in vitamins D and C, calcium, and protein to support bone and joint health.  3. Quit Smoking: Smoking can impair bone healing and decrease blood flow, worsening your condition.  4. Physical Activity: Engage in gentle exercises like swimming or walking, which are less stressful on your spine. Avoid high-impact activities.  5. Posture and Ergonomics: Practice good posture and adjust your workspace to be spine-friendly.  6. Regular Medical Checkups: Stay in close contact with your healthcare provider to monitor your condition and adjust treatments as necessary.  Implementing these changes can significantly help in managing your spondylodiscitis and improving your quality of life. |  |  |  |  |
| ChatGPT-4o:  To manage Spondylodiscitis, focus on supporting your recovery and preventing further complications through lifestyle changes:  1. Rest and Activity: Allow your body to heal by avoiding heavy lifting and intense physical activity. However, gentle movements like walking and stretching, guided by a physiotherapist, can help improve flexibility and strength.  2. Good Posture: Maintain proper posture when sitting and standing to reduce strain on your spine.  3. Nutrition: Eat a balanced diet rich in protein, vitamins (especially vitamin D), and minerals like calcium to support bone healing.  4. Quit Smoking and Limit Alcohol: Smoking impairs bone health and healing, while excessive alcohol can weaken the immune system.  5. Medication Adherence: Follow your doctor's prescribed antibiotic regimen carefully, as this is crucial for treating the infection.  Always consult your healthcare team before making significant lifestyle adjustments. |  |  |  |  |
| Gemini (Google Bard):  Spondylodiscitis, an infection of the intervertebral disc, can be managed with lifestyle changes alongside medical treatment.  Key lifestyle modifications include:   1. Rest: Avoid activities that aggravate pain, such as heavy lifting or strenuous exercise. Gradually increase activity as your condition improves. 2. Back Support: Use a supportive mattress and pillows to maintain proper spinal alignment. 3. Weight Management: Excess weight can put strain on your spine. Aim for a healthy weight through balanced diet and exercise. 4. Smoking Cessation: Smoking can delay healing and increase the risk of complications. 5. Stress Management: Stress can exacerbate pain. Explore relaxation techniques like meditation or deep breathing.   Remember, these lifestyle changes can complement your medical treatment plan. Always consult with your healthcare provider before making significant adjustments to your routine. |  |  |  |  |

Table 10: Can Spondylodiscitis be prevented?

| Question:  "Can Spondylodiscitis be prevented?" | excellent answer not requiring clarification | satisfactory, requiring minimal clarification | satisfactory, requiring moderate clarification | unsatisfactory, requiring substantial clarification |
| --- | --- | --- | --- | --- |
| ChatGPT-4:  Spondylodiscitis, an infection of the spinal discs and adjacent vertebrae, is challenging to prevent completely due to its varied causes, including bacteria entering the bloodstream and reaching the spine. However, certain strategies can reduce risk:  1. Prompt treatment of infections: Treating infections elsewhere in the body quickly can prevent bacteria from spreading to the spine.  2. Hygiene and Care after Procedures: Ensuring proper sterilization and post-operative care following surgeries or invasive procedures can minimize infection risks.  3. Health Management: For individuals with conditions like diabetes or those using immunosuppressants, maintaining optimal health and regular medical checkups are crucial to prevent infections.  While absolute prevention isn't always possible, these measures significantly lower the risk of developing spondylodiscitis. |  |  |  |  |
| ChatGPT-4o:  Spondylodiscitis, an infection of the spine's intervertebral discs and adjacent vertebrae, is difficult to prevent completely, but certain steps can reduce the risk. Prevention focuses on minimizing infection risks, particularly in people with weakened immune systems, diabetes, or prior spinal procedures. Key measures include:  1. Promptly Treating Infections: Early treatment of infections (like UTIs or skin infections) can help stop the spread to the spine.  2. Good Hygiene and Wound Care: For surgical patients, proper post-op wound care and infection control measures in hospitals are crucial.  3. Managing Chronic Conditions: Control of chronic conditions like diabetes and avoiding prolonged use of immunosuppressive drugs reduces vulnerability to infections.  4. Avoiding Intravenous Drug Use: This significantly decreases the risk of bacterial infections spreading to the spine.  While not always preventable, staying vigilant with health and infection control helps lower the chances of developing spondylodiscitis. |  |  |  |  |
| Gemini (Google Bard):  Yes, there are steps you can take to reduce your risk of spondylodiscitis. While it's not always preventable, especially in individuals with underlying health conditions like diabetes or immune system disorders, certain measures can help:   1. Maintain good hygiene: Practice proper handwashing and avoid contact with infected individuals. 2. Manage underlying health conditions: Regularly monitor and treat conditions like diabetes, HIV, and rheumatoid arthritis. 3. Avoid intravenous drug use: This practice can increase the risk of infections, including spondylodiscitis. 4. Take precautions during invasive procedures: Ensure that medical procedures are performed in sterile environments and by qualified healthcare professionals.   If you have concerns about your risk of spondylodiscitis, it's important to discuss these with your healthcare provider. They can provide personalized advice and recommendations based on your individual situation. |  |  |  |  |
