# Supplementary figures and images for: Publicly Accessible Large Language Model Responses to Frequently Asked Questions About Spondylodiscitis: Preliminary Expert Evaluation
Source: J Med Internet Res. 2026 Jul 16;28:e90364. doi: 10.2196/90364 (PMC13374826; doi:10.2196/90364)

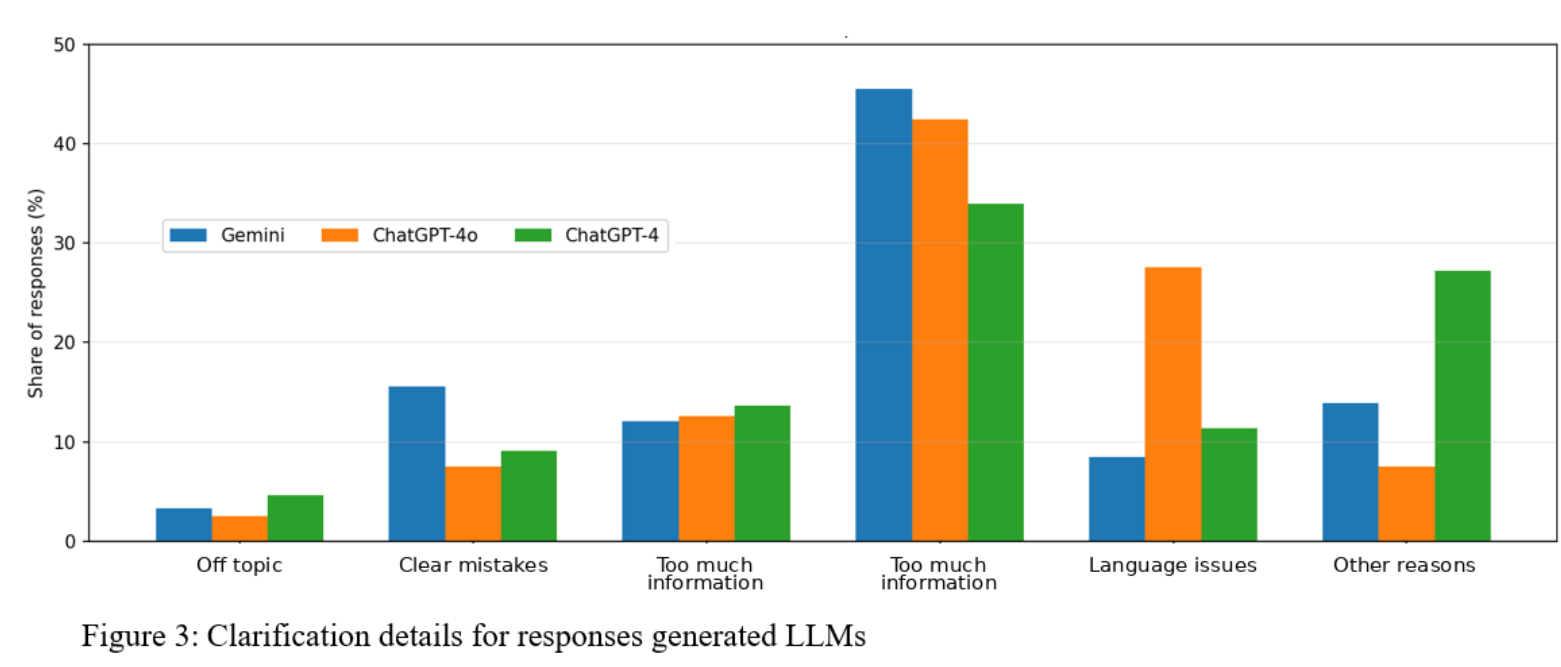

Supplement: Figure S1. [file jmir-v28-e90364-s003.png]
